# Literary Piracy

**Published:** 1858-11

**Authors:** 


*Literary Piracy.*—We extract from the B. & F. Medico-Chirurgical Review, the following remarkable instance of literary piracy:

*A Contribution to the Curiosities of Medical Literature.*—The French Society of Surgery has recently been subjected to a mystification which, to all but the persons immediately interested, may appear somewhat amusing, were it not that ethical principles are involved which forbid the rising smile. A work on stricture of the urethra was presented to the Société de Chirurgie, in December, 1856,* in which the author, Dr. Pró, claims the merit of having made, at the suggestion of M. Malgaigne, extensive personal investigations on the subject, especially in the pathological museums of London. What he terms the *travail spécial, qui forme pour ainsi dire le corps du mémoire*, occupying about twenty-five out of the one hundred and twenty-two pages of the work, is a verbatim translation of a part of Mr. Henry Thompson's well-known work,† which received the Jacksonian prize for the year 1852. No acknowledgment of the source from which Dr. Pró has obtained such valuable assistance is given, and the translation is so well done, that we can understand how the referees of this society, who reported well on the memoir, and who could not be familiar with Mr. Thompson's work, should have been deceived into the belief that the production before them was a *bonâ fide* work of the professed author. It appears that Dr. Pró is not a Frenchman, and we should imagine that he must be unacquainted with the facilities of intercourse, personal and literary, which exists throughout Europe, but especially between France and Great Britain, otherwise he could not have ventured upon such an act of plagiarism. The assurance of subsequent detection would have been a sufficient check upon him. We much regret, for the sake of the distinguished men who formed the commission to

---

* Mémoire sur l'Anatomie Pathologique des Rétrécissements de l'Urèthre. Par Jose Pró, Docteur en Médecine de la Faculté de Paris. Paris, 1856. pp. 122.

† The Pathology and Treatment of Stricture of the Urethra, both in the Male and Female. By Henry Thompson, F. R. C. S. London, 1854.

which the book was referred for examination, that they should have been led to regard the work as the result of Dr. Pró's labors, whereas the most important parts belong to Mr. Henry Thompson. We can well conceive their annoyance on the circumstances being pointed out to them. At the same time, it is proper that such an act of literary piracy should be loudly proclaimed, and that a well-earned reputation should not suffer through a maudlin sentimentality in favor of the culprit. We understand that steps have already been taken to prevent the recommendation of the commission from taking effect—viz., that the author should be made a corresponding member of the Sociéte de Chirurgie.

